# Clinical significance and potential mechanisms of *miR-223-3p* and *miR-204-5p* in squamous cell carcinoma of head and neck: a study based on TCGA and GEO

**DOI:** 10.1515/med-2020-0146

**Published:** 2020-08-03

**Authors:** Lei Zhao, Congzhe Tian, Erbin Xiao, Jinduo Du, Jingwei Liang, Xianghong Chen, Weiwei Chi

**Affiliations:** Department of Otorhinolaryngology, The Affiliated Hospital of Hebei University, Baoding, No. 212 Yuhua Road, Hebei Province, 071000, China; Department of Otorhinolaryngology, The First Hospital of Hebei Medical University, Shijiazhuang, Hebei Province, 050031, China

**Keywords:** head and neck tumor, miRNA, *miR-223-3p*, *miR-204-5p*, SCCHN

## Abstract

**Objective:**

To explore the clinical significance and mechanisms of altered miRNAs in squamous cell carcinoma of head and neck (SCCHN) and provide references for SCCHN diagnosis and prognosis.

**Method:**

Differential expressed miRNAs (DEMs) in SCCHN were screened through gene expression omnibus (GEO) DataSets and verified by the cancer genome atlas (TCGA) database. Next, the overall survival analysis, receiver operating characteristics, and clinical correlation analysis were adopted to filter the miRNAs with diagnostic and prognostic values. Finally, functional enrichment analyses were conducted for inquiring into the mechanisms of miRNAs.

**Results:**

Total 103 DEMs (*p* < 0.05, fold change ≥ 2) in SCCHN were screened out from GSE124566. Partly, the expression levels of the selected (12/17) miRNAs were verified by TCGA. Followed, of the 12 miRNAs, two miRNA expression levels were associated with the overall survival, and five miRNAs showed diagnostic values (AUC ≥ 0.85). Besides, *miR-223-3p* and *miR-204-5p* expression levels were correlated to certain clinical features. Epithelial–mesenchymal transition (EMT) related biological process and energy metabolism controlling related *AMPK* signaling pathway might mediate the roles of *miR-223-3p* and *miR-204-5p*, respectively.

**Conclusion:**

With diagnostic and prognostic values, *miR-223-3p* and *miR-204-5p* may be involved in the progression of SCCHN through EMT-related biological process and energy balance related *AMPK* signaling pathway, respectively.

## 1 Introduction

As a heterogeneous malignant tumor, squamous cell carcinoma of head and neck (SCCHN) originates from lip, oral cavity, larynx, and pharynx, and is responsible for the high morbidity and mortality of malignant tumors worldwide [1]. According to GLOBOCAN 2018, approximately 8,34,860 new SCCHN cases registered and 4,31,131 cases died of it worldwide in 2018 [2]. Apart from the complicated anatomical features, an insufficient understanding of the pathogenesis and the absence of effective target therapy are also the pivotal reasons for the poor diagnosis and prognosis of SCCHN [1,3,4]. Hence, exploring the critical regulatory mechanisms and identifying the key effect molecules in the pathogenesis and progression of SCCHN are extensively concerned by researchers, and numerous achievements have been made, covering the DNA damage repair [5], effects of exosomes on tumor angiogenesis [6], roles of long non-coding in epithelial–mesenchymal transition (EMT) [7], positive or negative regulation of microRNAs (miRNAs) [8,9,10], and other aspects.

Here, the present study focused on exploring the regulatory functions of miRNAs in SCCHN for its pivotal biological activity. Previous studies have documented that miRNAs exerted significant biological effects on the occurrence and development of SCCHN. Certain miRNAs suppress SCCHN cellular growth, migration, and invasion ability, and reverse the progression of EMT through diverse mechanisms. For instance, the overexpression of the downregulated *miR-4282* retards the progression of oral squamous cell carcinoma by regulating the *LIN28B/ZBTB2* pathway in gain-of-function assays [11]. The downregulated *miR-204-5p* inhibits the SCCHN progression by forming a regulatory complex with *SNAI2*/*SUZ12*/*HDAC1*/*STAT3* [12]. Moreover, the abnormally expressed miRNAs influence the cellular resistance against the radiotherapy or chemotherapy through *PI3K-Akt* signaling pathway, *miR-936*/*GPR78* or *miR-372*-*ZBTB7A*-*TRAIL-R2* pathway in SCCHN [13,14,15]. Even altered miRNAs could attenuate the angiogenesis and metastasis [16], genomic stability [17], and immune evasion [9]. The same miRNA could regulate multiple mRNAs, such as the inhibitory effect of *miR-129* on *CDK4*, *CDK6*, and *MDM2* [18], and multiple miRNAs could also regulate the same mRNA simultaneously, such as the regulation of *miR-21* and *miR-499* on *PDCD4* [19], which is another embodiment of complexity and diversity of miRNAs’ regulatory mechanisms. Apart from the complicated and varied effect mechanisms, pan-cancer character is also responsible for its rising concern. For the same miRNA, it may be involved in various biological behaviors of multiple cancers, which accelerated the widespread development of the related studies, such as the regulatory effects of *miR-129-5p* on liver cancer, rectal adenocarcinoma, nasopharyngeal carcinoma, and prostate cancer [20,21,22,23].

Based on the above-mentioned points, the present study concentrated on exploring the altered miRNAs and corresponding roles in SCCHN through mining public databases and re-analyses, hoping to provide valuable reference for further research studies on SCCHN. After a series of bioinformatics analyses, the present study screened out 103 ectopic expressed miRNAs in SCCHN and partly verified by the cancer genome atlas (TCGA) database. Further detailed analyses indicated that *miR-223-3p* and *miR-204-5p*, with potential diagnostic and prognostic values, might involve in the occurrence and development of SCCHN through the mediation of EMT-related biological process and energy metabolism controlling related *AMPK* signaling pathway.

## 2 Materials and methods

### 2.1 Ethics, consent, and permission

The present study was authorized by the Ethics Committee of the Affiliated Hospital of Hebei University (No. HDFY-LL-2020-008). Written consent and permission from the patients were not needed.

### 2.2 Expression profiles’ mining and screening

Gene expression omnibus (GEO) DataSets (http://www.ncbi.nlm.nih.gov/geo/), a public and open accessible database for the expression profile storage [24], was applied to mine SCCHN-related miRNA expression profiles based on retrieval terms: (miRNA OR microRNA) AND (HNSCC OR SCCHN OR “head and neck squamous cell carcinoma” OR “squamous cell carcinoma of head and neck”). Screening criteria include: (1) study type: non-coding RNA profiling by array; (2) tissue: paired cancerous and adjacent non-cancerous samples; (3) retrieval duration: from database creation to retrieval of data on February 25, 2020.

### 2.3 Differential expressed miRNA screening

GEO2R (https://www.ncbi.nlm.nih.gov/geo/info/geo2r.html), an implanted online tool for comparing differential expressed genes in two or more groups of samples [25], was applied to screen the differential expressed miRNAs (DEMs) in the included GEO Series with the default parameters: Benjamini & Hochberg false discovery rate and *p* < 0.05. And then, the DEMs were further filtered manually according to adjustment of *p* < 0.05 and fold change ≥2.

### 2.4 Acquisition of the miRNA expression profile for verification

The TCGA database (https://www.cancer.gov/tcga), a database for free and open access to genome, transcriptome, and other data [26], was adopted for the acquisition of a miRNA expression profile. And Genomic data commons data portal (https://portal.gdc.cancer.gov/), a data-driven platform for searching and downloading cancerous data [27], was used to implement the acquisition process according to the user’s guide (https://docs.gdc.cancer.gov/).

### 2.5 miRNA targets prediction and filtering

TargetScanHuman 7.2 (http://www.targetscan.org/vert_72/), a free and open access online database for miRNA targets’ prediction [28], miRDB (http://mirdb.org/), an online database to predict miRNA targets based on thousands of high-throughput sequencing experiments [29], and miRWalk (http://mirwalk.umm.uni-heidelberg.de/), an online database for predicting miRNA targets with a machine learning algorithm including the experimental verification of miRNA–target interactions [30], were applied for predicting potential miRNA targets. Further, Venn diagrams (http://bioinformatics.psb.ugent.be/beg/tools/venn-diagrams), an online tool for calculating the intersections of multiple datasets, was used for obtaining the intersections of the above three databases for selected miRNAs.

### 2.6 Functional annotation for miRNA targets

The database for annotation, visualization and integrated discovery (DAVID) v6.8 (https://david.ncifcrf.gov/), an online resource for providing comprehensively functional annotations [31], was used for exploring the potential mechanisms exerted by interested miRNAs. The analysis was completed following the typical analysis flow in the user’s manuals (https://david.ncifcrf.gov/content.jsp?file=functional_annotation.html). The detailed parameters were as follows: target mRNAs as the gene list for loading, official gene symbol as the retrieval identifier, and homo sapiens as the background.

### 2.7 Statistical analysis

Results were analyzed with SPSS 22.0 software (IBM SPSS, USA). Independent sample *t*-test or Mann–Whitney *U* test was used for measurement data and chi-square test was used for enumeration data. Related diagrams were completed by GraphPad Prism 7 software (GraphPad Software Inc., USA). A value of *p* < 0.05 means a statistic difference.

## 3 Results

### 3.1 SCCHN-related miRNA expression profiles in GEO DataSets

**Figure 1:**
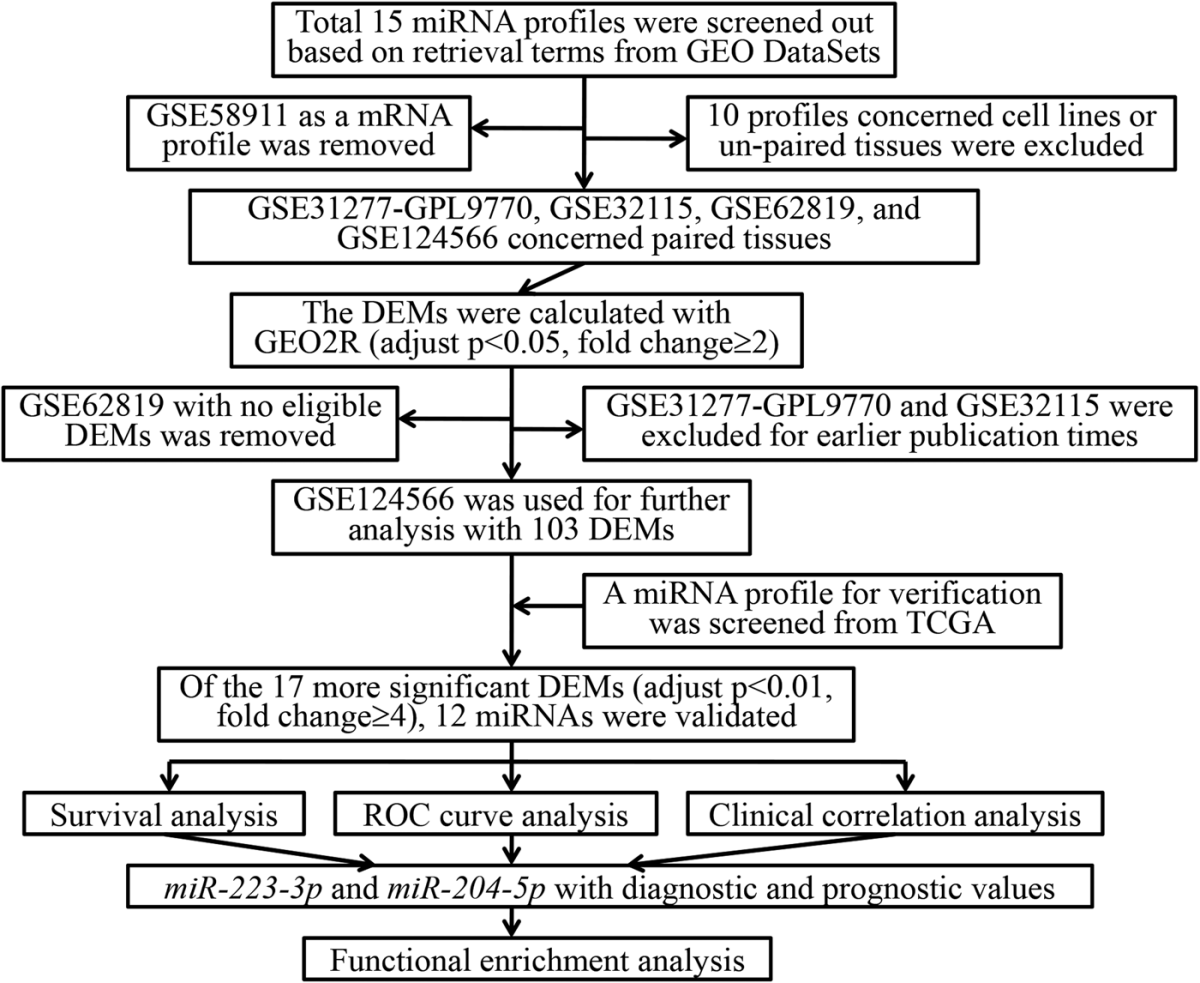
Flowchart for analyses. GEO, gene expression omnibus , DEMs, differential expressed miRNAs, TCGA, the cancer genome atlas, ROC, receiver operating characteristic.

By searching the miRNA expression profiles of SCCHN in GEO DataSets based on retrieval terms, 15 profiles matching the retrieval terms were screened out. After a detailed review of the 15 profiles, some of them were excluded, according to the screening criteria. Of them, GSE58911 dataset was first eliminated as a de facto mRNA expression profile. Another five profiles (GSE10301, GSE25524, GSE32176, GSE33196, and GSE79369) concerning SCCHN cell lines and three profiles (GSE11163, GSE34496, and GSE92595) concerning unpaired SCCHN tissues were also excluded, due to dissatisfaction with the criteria. In addition, GSE33232 and GSE79372, integrated datasets composed of four subseries separately, were further eliminated due to cell lines and unpaired tissue sources. Moreover, GSE31277, also a dataset, concerned cell lines and unpaired tissues and was composed of three platforms, which could be extracted using a miRNA expression profile of 14 paired tissues based on platform GPL9770, namely, GSE31277-GPL9770. Subsequently, GSE31277-GPL9770 and three other datasets based on paired tissues (GSE32115, GSE62819, and GSE124566) were enrolled in subsequent analyses (Figure 1).

### 3.2 Distribution of DEMs in each included dataset

For each included dataset, the DEMs were calculated with GEO2R and further filtered according to adjustment of the value of *p* < 0.05 and fold change ≥2. Subsequently, total 76, 103, and 29 DEMs were acquired from GSE32115, GSE124566, and GSE31277-GPL9770, respectively. Notably, after screening by adjusting the value of *p* < 0.05, no eligible DEMs were screened out in GSE62819. Hence, GSE62819 was further eliminated from the present study. Further analysis indicated that there was no intersection among the remaining three datasets, but only a few DEMs that co-existed in two datasets. Considering that the amount of DEMs and integrity of annotation databases were affected by the time of publication, GSE32115 (published in 2011) and GSE31277-GPL9770 (published in 2014) were further removed from subsequent analyses. Ultimately, the recently published dataset GSE124566 (published in 2020) was selected for subsequent analyses with 103 DEMs (Figure 1).

### 3.3 Details of DEMs in GSE124566

**Figure 2:**
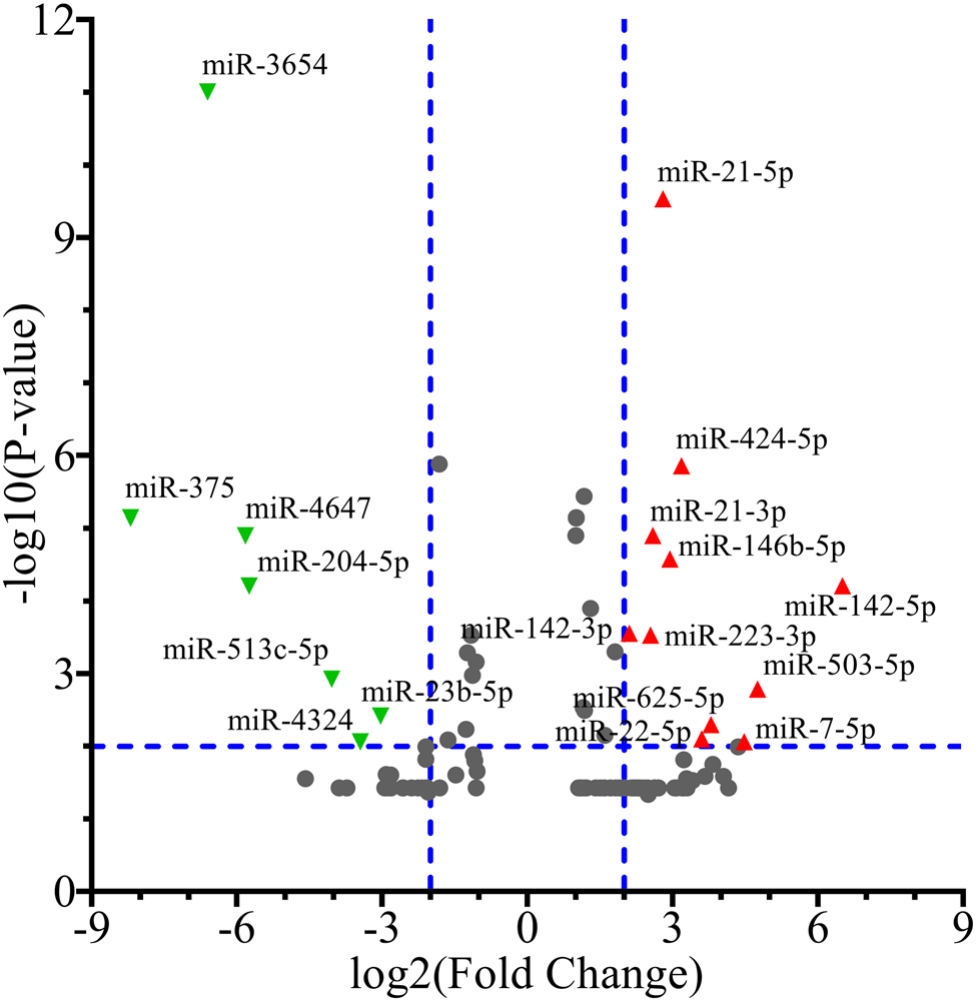
Differential expressed miRNAs of GSE124566. Red triangles represent the 11 overexpressed miRNAs and green triangles represent the seven downexpressed miRNAs in cancerous tissues of SCCHN, respectively. Gray dots represent miRNAs with no significant difference (adjustment of *p* < 0.01 and fold change ≥4). SCCHN, squamous cell carcinoma of head and neck.

Through the preliminary screening criteria (adjustment of *p* < 0.05 and fold change ≥2), total 103 DEMs (63 overexpressed and 40 downexpressed miRNAs) were screened out from GSE124566. For acquiring the more significant DEMs in GSE124566, the screening criteria were further narrowed down to adjust *p* < 0.01 and fold change ≥4. Total 18 more significant DEMs (11 overexpressed and 7 downexpressed miRNAs) were screened out and used for subsequent analyses (Figure 2).

### 3.4 SCCHN-related miRNA expression profile in TCGA

**Figure 3:**
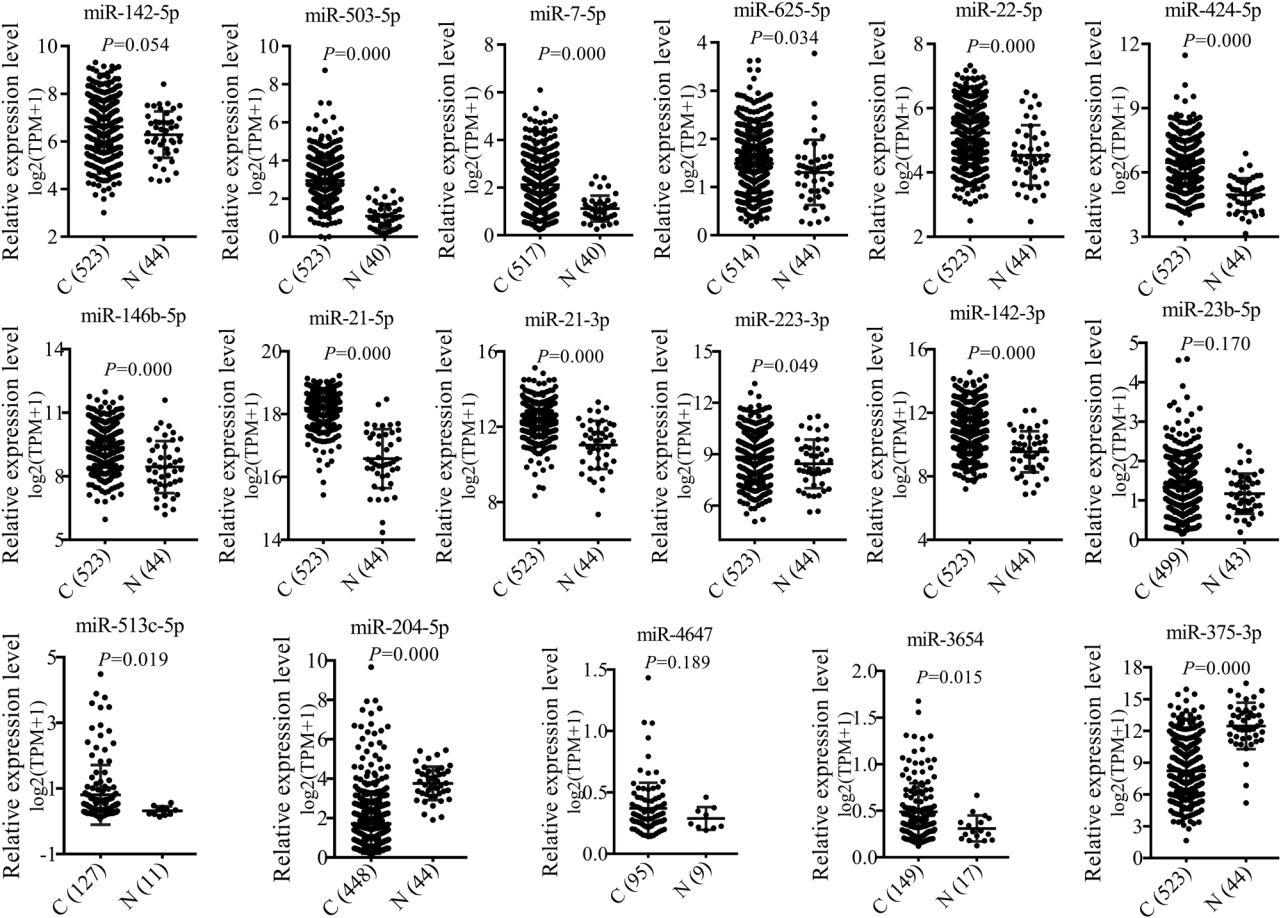
Expression levels of miRNAs in SCCHN based on TCGA database. *X*-axis represents the types of tissues, C, cancerous tissues, N, normal tissues. The numbers in parentheses represent the number of samples. Due to the absence of some miRNA expression values in expression profile data, the sample sizes are unequal for different miRNAs. *Y*-axis represents the relative expression levels of miRNAs in SCCHN tissues. TPM, transcripts per million. A value of *p* < 0.05 means significant difference.

For verification, the miRNA expression levels of GSE124566 in SCCHN, a different miRNA expression profile, were acquired from TCGA, which consists of 524 cancerous tissues and 45 normal tissues of SCCHN and includes 2,242 miRNAs (Query strategy: Project Id IS TCGA-HNSC AND Access IS open AND Data Category IN (clinical transcriptome profiling) AND Data Format IS txt AND Data Type IS miRNA Expression Quantification). Based on the TCGA miRNA expression profile, the 18 DEMs of GSE124566 were well validated in expression tendency. Of the 18 miRNAs, *miR-4324* is not included in the TCGA miRNA expression profile. For the remaining 17 miRNAs, total 12 miRNA expression trends in TCGA expression profile were consistent with GSE124566 (Figure 3). Nevertheless, *miR-142-5p*, *miR-23b-5p*, and *miR-4647* showed no significant difference in the expression trend between cancerous and normal tissues. Even *miR-513c-5p* and *miR-3654* showed an opposite expression tendency to GSE124566.

### 3.5 Relationships between the expression levels of 12 validated miRNAs and overall survival times

**Figure 4:**
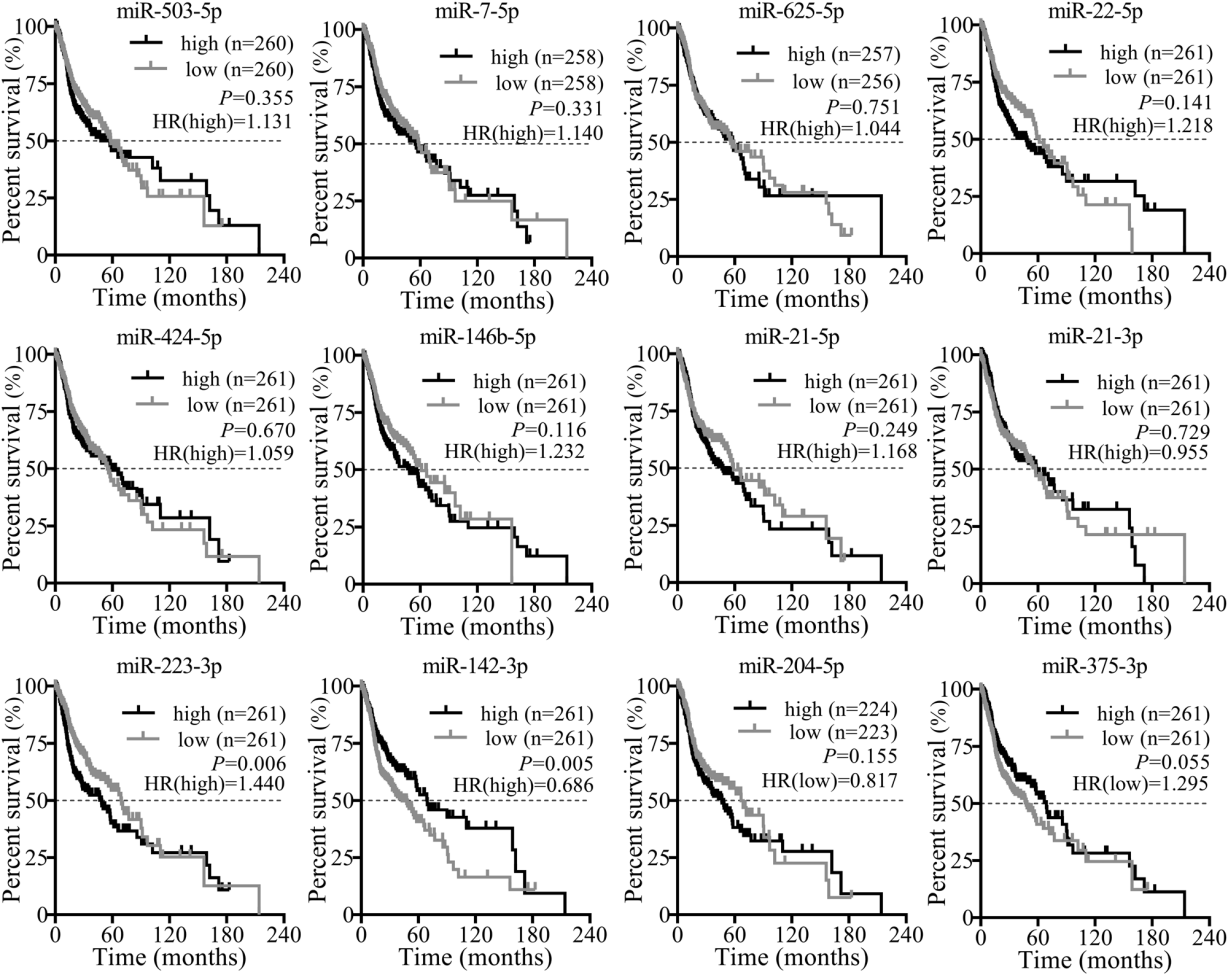
Overall survival analyses for differential expressed miRNAs. *X*-axis represents the overall survival time and *Y*-axis represents the survival rates at different times. High means the miRNA expression levels were higher than the median of total miRNA expression levels, while low the opposite. HR: hazard ratio. HR (high) means the hazard ratio of high expression group to low expression group, while HR (low) the opposite. A value of *p* < 0.05 and HR > 0.5 mean significant difference.

Based on the clinical data and miRNA expression levels of TCGA, survival analyses showed that most of the 12 validated miRNAs correlated with overall survival times with high hazard ratios (HR). However, merely *miR-223-3p* and *miR-142-3p* showed significant differences with lower *p*-values (*p* < 0.05) (Figure 4). Inconsistently, for an overexpressed *miR-142-3p* in cancerous tissues, it seems to present that patients with high expression levels of *miR-142-3p* may have a long survival period. Consequently, survival analyses indicated that patients with high-level *miR-223-3p* have shorter survival times than patients with low expression levels (*p* = 0.006, HR = 1.440) (Log-rank test).

### 3.6 Diagnostic values of the 12 validated DEMs

**Figure 5:**
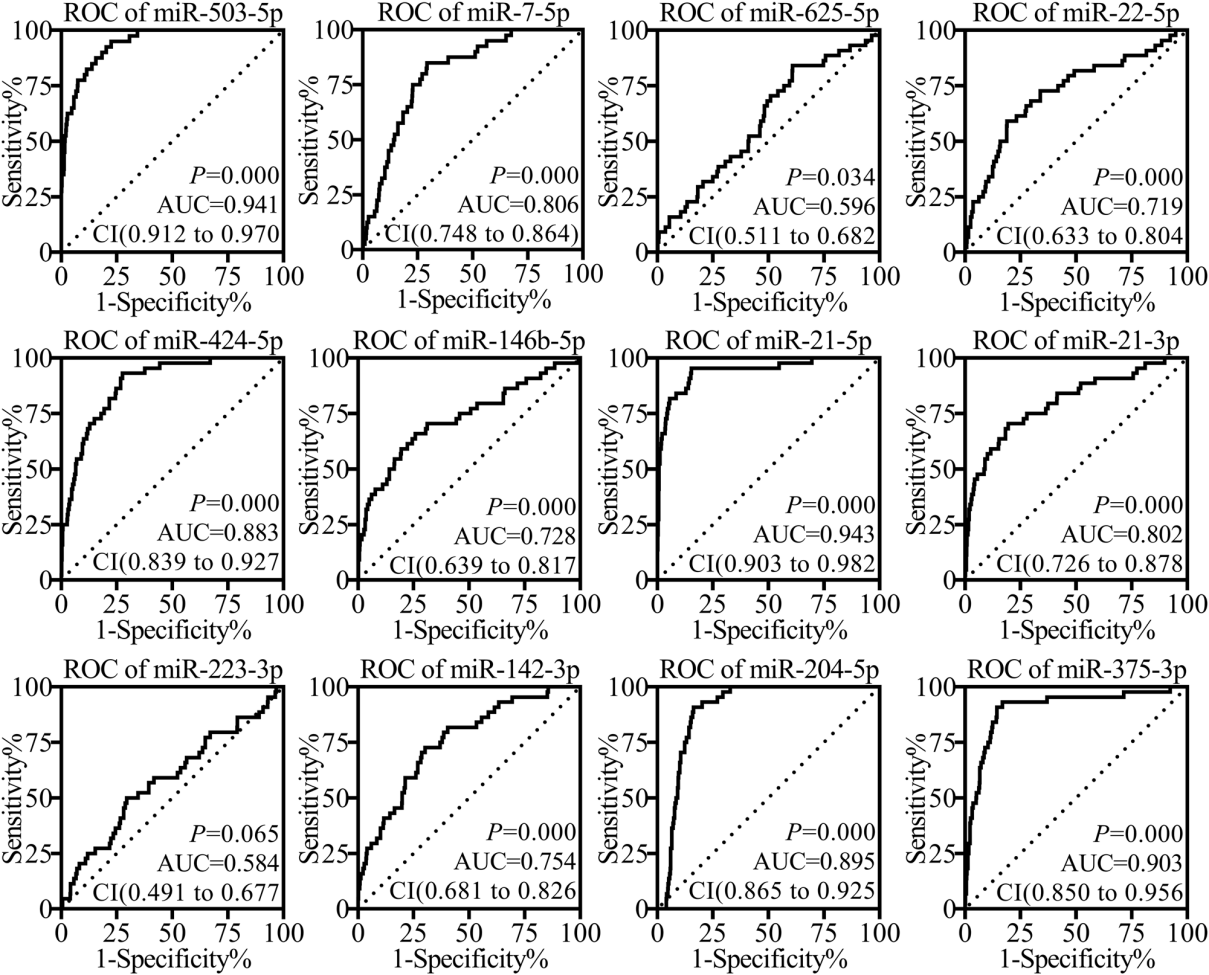
Receiver operating characteristic curves of differential expressed miRNAs. ROC, Receiver operating characteristic, AUC, area under ROC curve. CI, confidence interval. A value of *p* < 0.05 and AUC ≥ 0.85 mean effective diagnostic values.

Through a receiver operating characteristic (ROC) curve analyzing the diagnostic values of 12 validated DEMs, the results presented that five out of 12 miRNAs may have potential diagnostic values with high area under ROC curves (AUC) (AUC ≥ 0.85, *p* < 0.05), including *miR-503-5p*, *miR-424-5p*, *miR-21-5p*, *miR-204-5p*, and *miR-375-3p* (Figure 5).

### 3.7 Comparison of the expression levels of miRNAs with diagnostic or prognostic values in different clinical feature groups

For exploring the associations between the miRNA expression levels and clinical features, the expression levels of miRNAs (*miR-223-3p*, *miR-503-5p*, *miR-424-5p*, *miR-21-5p*, *miR-204-5p*, and *miR-375-3p*) in different groups with distinct clinical features were compared and analyzed. Analyses showed that *miR-223-3p*, *miR-503-5p*, and *miR-424-5p* expression levels varied with different genders (*p* < 0.05). Besides, *miR-223-3p* expression levels were changed in different clinical stages (*p* = 0.026) and *miR-204-5p* expression levels were also distinctive in different differentiation degrees (*p* = 0.036). Added, specimens with smoking or without smoking showed different *miR-503-5p* expression levels (*p* = 0.000) (Table 1).

**Table 1:** Comparison of miRNA expressions in different clinical features

		Gender		Age (years)		Drinking		Smoking		Stage		Differentiation		Lymph node metastasis	
		Male	Female	<61	≥61	No	Yes	No	Yes	I–II	III–IV	High-medium	Low	No	Yes
miR-223-3p	High (*n*)	178	84	119	142	83	174	62	195	66	196	195	64	131	118
	Low (*n*)	204	57	138	123	81	176	59	194	45	216	175	68	114	141
	*χ*2		6.938		2.767		0.036		0.046		4.942		0.693		3.151
	*p*		**0.008**		0.096		0.850		0.831		**0.026**		0.405		0.076
miR-503-5p	High (*n*)	180	81	138	122	83	174	82	175	55	206	192	57	116	133
	Low (*n*)	200	60	118	142	81	174	39	212	56	204	176	75	127	126
	*χ*2		4.178		3.078		0.017		18.750		0.017		3.142		0.655
	*p*		**0.041**		0.079		0.898		**0.000**		0.897		0.076		0.418
miR-424-5p	High (*n*)	180	82	131	130	74	181	70	188	60	202	180	71	120	134
	Low (*n*)	202	59	126	135	90	169	51	201	51	210	190	61	125	125
	*χ* ^2^		5.017		0.192		1.941		3.348		0.883		1.028		0.383
	*p*		**0.025**		0.662		0.164		0.067		0.347		0.311		0.536
miR-21-5p	High (*n*)	194	68	125	137	78	179	63	193	54	208	173	74	121	132
	Low (*n*)	188	73	132	129	86	171	58	196	57	204	197	58	124	127
	*χ* ^2^		0.270		0.429		0.573		0.222		0.118		3.370		0.125
	*p*		0.604		0.512		0.449		0.638		0.731		0.066		0.723
miR-204-5p	High (*n*)	155	69	103	120	75	145	55	163	57	167	147	67	114	103
	Low (*n*)	172	52	117	107	69	151	52	167	43	181	167	48	100	115
	*χ* ^2^		3.272		1.633		0.372		0.130		2.523		4.411		1.567
	*p*		0.071		0.201		0.542		0.718		0.112		**0.036**		0.211
miR-375-3p	High (*n*)	193	69	131	131	74	173	55	198	58	204	187	67	130	119
	Low (*n*)	189	72	126	134	90	167	66	191	53	208	183	65	115	140
	*χ* ^2^		0.104		0.124		1.469		1.095		0.262		0.002		2.550
	*p*		0.747		0.725		0.226		0.295		0.609		0.966		0.110

Samples were divided into high expression levels and low expression levels according to the median of expression levels for each miRNA. Bold represents significant difference. Sixty-one years represent the median age.

### 3.8 Prediction of target mRNAs and functional enrichment analyses for *miR-223-3p* and *miR-204-5p*

Comprehensively considering survival analyses, ROC curve and miRNA differential expression levels in distinct clinical feature groups, *miR-223-3p* and *miR-204-5p* were selected for further analyses to filter the significant miRNAs in SCCHN. Followed, the target mRNAs of *miR-223-3p* and *miR-204-5p* were predicted by TargetScanHuman 7.2, miRDB, and miRWalk separately. The intersection mRNAs of the three databases (103 mRNAs for *miR-223-3p* and 292 mRNAs for *miR-204-5p*) were used for subsequent analyses. Afterwards, functional enrichment analyses of intersection mRNAs showed that *miR-223-3p* or *miR-204-5p* may be involved in multiple biological processes, molecular functions, and signaling pathways, respectively. Analyses illustrated that *miR-223-3p* may participate in multiple biological processes (GO: 0001837 ∼ epithelial-to-mesenchymal transition and GO: 0030177 ∼ positive regulation of *Wnt* signaling pathway) and molecular functions (GO: 0046322 ∼ SMAD binding and GO: 0008134 ∼ transcription factor binding), which indicated that *miR-223-3p* may be involved in the regulation of EMT. Analyses also illustrated that *miR-223-3p* and *miR-204-5p* may be involved in the process of energy anabolism and catabolism mediated by the same signaling pathway (hsa04152: *AMPK* signaling pathway).

## 4 Discussion

The expression profile is a composite of multifarious valuable information, for instance, the difference in overall gene expression trend between the experimental group and the control group, the distribution of differential expressed genes in the experimental group or the control group, and the difference of a specific gene among different specimens. Since the above-mentioned characteristics, re-analyses of gene expression profiles acquired more valuable and significant references for further research studies [32,33,34,35]. Hitherto, numerous profiles, involving coding and non-coding RNA expression profiles, genome variation profiles, protein profiles, and methylation profiles, have been published and reserved in GEO [24], TCGA [26], or Oncomine (https://www.oncomine.org/) [36], which could be re-analyzed. A study elaborated the significant prognostic values of multiple genes for SCCHN patients through re-analyses based on TCGA and GEO profiles [37]. Our previous studies also completed the screening of SCCHN-related hub genes through re-analyses of mRNA expression profiles [33], and mined a significantly overexpressed long-non-coding RNA *LINC00668* for further study of non-coding RNA profiles [34]. Here, the present study further explored the miRNAs with diagnostic and prognostic values in SCCHN with the method of re-analyses of miRNA expression profiles.

After rigorous screening, a recently published miRNA expression profile of SCCHN (GSE124566) was included in the present analyses. The original research study, which published the dataset GSE124566, proposed that total 43 deregulated miRNAs (fold change >2, adjusted *p* < 0.01) were screened out from included SCCHN tissue specimens [12]. In the present study, total 18 DEMs were screened out from GSE124566 (fold change ≥4, adjusted *p* < 0.01), all of which were included in the above-mentioned 43 deregulated miRNAs. Of the 18 miRNAs, 12 miRNA expression trends were verified by TCGA, including the downregulated *miR-204-5p* in cancerous tissues, a proved tumor suppressor by the original research study [12]. The consistency between the re-analyses and the original study further confirmed the reliability of the present study and promoted the progression of the study. Followed survival analyses presented that the high levels of *miR-223-3p* were related to the poor prognosis of SCCHN patients, which indicated that *miR-223-3p* may be a prognostic marker for SCCHN. In addition, the survival analyses also suggested that the patients with high expression levels of *miR-142-3p* presented significantly long survival times. This phenomenon seems to embody a protective effect of *miR-142-3p* on SCCHN patients, but this effect is contrary to the overexpression of *miR-142-3p* in cancerous tissues. This result needs a thorough study to explore the definite role of *miR-142-3p* in SCCHN. Certain miRNAs are helpful for cancer diagnoses [38]. In view of this, the present study distinguished five miRNAs with possible diagnostic values, which consisted of *miR-503-5p*, *miR-424-5p*, *miR-21-5p*, *miR-204-5p*, and *miR-375-3p*. All of the five miRNAs are worthy of further study on cancerous diagnosis for identifying an effective tumor marker for diagnosis. Of the above-mentioned six miRNAs with prognostic or diagnostic values, *miR-223-3p* and *miR-204-5p* presented significant distinct expression levels in different clinical stages or tissue differentiation, respectively. Notably, for *miR-204-5p*, the original study indicated that the patients with high expression levels of *miR-204-5p* showed higher clinical stages and positive neck lymph node metastasis [12]. However, apart from tissue differentiation degrees, the present study has not been able to ascertain the correlations between *miR-204-5p* expression levels and clinical stages or lymph node metastasis. This inconsistency may be related to the heterogeneity of tumor tissues, and it is likely to be eliminated following the implementation of follow-up studies. However, the prognostic value of *miR-204-5p* and its correlation with clinical features suggested that *miR-204-5p* may be an effective tumor marker or target for diagnostic and prognostic evaluation or therapy, which has been elaborated and confirmed in the original research study [12]. The present analyses also indicated that the *miR-503-5p* expression levels were related to smoking history, an agreed risk factor for the occurrence and development of SCCHN [39]. This finding suggested that *miR-503-5p* may play an unknown role in the effect of tobacco on SCCHN.

In general, miRNAs exert diverse regulatory roles through target differential mRNAs [11,14,16,22,40], same for *miR-223-3p* and *miR-204-5p*. Considering the potential clinical significance of *miR-223-3p* and *miR-204-5p*, it is necessary and worthy to define the possible targets for providing reference for the followed study. Therefore, TargetScanHuman 7.2, miRDB, and miRWalk jointly predicted the targets of *miR-223-3p* and *miR-204-5p*, respectively. The joint prediction of multiple databases greatly heightened the dependability of target mRNAs and the feasibility of subsequent study. Functional analyses based on target mRNAs speculated that *miR-223-3p* could be involved in the progression of SCCHN through EMT, a confirmed regulatory mechanism for the development of SCCHN [41,42,43]. It may be a novel angle to elucidate the pathogenesis of SCCHN from the perspective of *miR-223-3p* regulating the EMT. Analyses also suggested that *miR-223-3p* and *miR-204-5p* may affect the energy balance of SCCHN through *AMPK* signaling pathway, a proved regulatory signaling pathway for the energy metabolism of tumors [44,45,46,47]. Energy metabolism plays important roles in cancer progression and the regulation of energy metabolism balance may become one of the effective anti-tumor treatment methods [48,49].

The present study screened out the altered 18 miRNAs in SCCHN through a series of analyses and identified the six miRNAs (*miR-223-3p*, *miR-503-5p*, *miR-424-5p*, *miR-21-5p*, *miR-204-5p*, and *miR-375-3p*) with significant prognostic or diagnostic values. Especially for *miR-223-3p* and *miR-204-5p*, the present analyses speculated that *miR-223-3p* may modulate the progression of SCCHN through the mediation of EMT. Meanwhile, *miR-223-3p* and *miR-204-5p* may also involve in the regulation of cancer energy metabolism through *AMPK* signaling pathway. It is worthy to further explore the potential molecular mechanisms for them, in consideration of the prognostic, diagnostic, and clinical significance.

## 5 Abbreviations

AUC: area under ROC curve
DAVID: The Database for Annotation, Visualization and Integrated Discovery
DEMs: differential expressed miRNAs
EMT: epithelial–mesenchymal transition
GDC: Genomic Data Commons
GEO: Gene Expression Omnibus
HR: hazard ratio
miRNAs: microRNAs
ROC: receiver operating characteristic
SCCHN: squamous cell carcinoma of head and neck
TCGA: The Cancer Genome Atlas
